# Navigating the Data Deluge at the SLS 2.0 Macromolecular Crystallography (MX) Beamlines

**DOI:** 10.1063/4.0000929

**Published:** 2025-10-27

**Authors:** Jiaxin Duan, Guillaume Gotthard, Vincent Olieric, Max Burian, Ludmila Leroy, Camilla Larsen, Meitian Wang, Filip Leonarski

**Affiliations:** 1 PSI, Villigen, Aargau, Switzerland; 2 DECTRIS, Baden, Aargau, Switzerland

## Abstract

The newly upgraded SLS 2.0 as a 4^th^ generation synchrotron delivers unprecedented brilliance, enabling exploration of biological structures at vastly improved timescales and throughput. Our upgraded Macromolecular crystallography (MX) beamlines aim to support cutting-edge experiments including high-throughput fragment screening (FFCS),^1^ automated data collection,^2^ room temperature^3^, time-resolved serial crystallography,^4^ and X-ray scattering tensor tomography (SAS-TT)^5^—all generating massive data volumes requiring efficient management solutions.

We are experimenting with different implementations including a multi-tiered approach utilizing HW-accelerated edge servers,^6^ PSI’s high- performance computing infrastructure, and DECTRIS CLOUD capabilities. The data processing pipelines operate both locally and remotely to support ongoing pilot experiments that will provide valuable insights into the performance and usability of these pipelines during user operation. This presentation will deliver firsthand experience from our active beamline commissioning in the beginning of SLS 2.0, sharing emerging strategies for handling high data rates. We will present our data acquisition, data processing and data reduction strategies. In addition, we will discuss the benefits and the challenges of our new data management systems. By sharing our current journey implementing these systems, we aim to contribute valuable perspectives to the MX community navigating similar challenges with increasing data volumes.
